# Identification of long non-coding RNAs involved in floral scent of *Rosa hybrida*

**DOI:** 10.3389/fpls.2022.996474

**Published:** 2022-10-04

**Authors:** Shaochuan Shi, Shiya Zhang, Jie Wu, Xintong Liu, Zhao Zhang

**Affiliations:** ^1^ Vegetable Research Institute, Shandong Academy of Agricultural Science, Jinan, China; ^2^ Beijing Key Laboratory of Development and Quality Control of Ornamental Crops, Department of Ornamental Horticulture, China Agricultural University, Beijing, China

**Keywords:** *Rosa*, floral scent, lncRNA, terpenoids, phenylpropanoids, benzenoids, fatty acid derivatives

## Abstract

Long non-coding RNAs (lncRNAs) were found to play important roles in transcriptional, post-transcriptional, and epigenetic gene regulation in various biological processes. However, lncRNAs and their regulatory roles remain poorly studied in horticultural plants. Rose is economically important not only for their wide use as garden and cut flowers but also as important sources of natural fragrance for perfume and cosmetics industry, but presently little was known about the regulatory mechanism of the floral scent production. In this paper, a RNA-Seq analysis with strand-specific libraries, was performed to rose flowers in different flowering stages. The scented variety ‘Tianmidemeng’ (*Rosa hybrida*) was used as plant material. A total of 13,957 lncRNAs were identified by mining the RNA-Seq data, including 10,887 annotated lncRNAs and 3070 novel lncRNAs. Among them, 10,075 lncRNAs were predicted to possess a total of 29,622 target genes, including 54 synthase genes and 24 transcription factors related to floral scent synthesis. 425 lncRNAs were differentially expressed during the flowering process, among which 19 were differentially expressed among all the three flowering stages. Using weighted correlation network analysis (WGCNA), we correlate the differentially-expressed lncRNAs to synthesis of individual floral scent compounds. Furthermore, regulatory function of one of candidate lncRNAs for floral scent synthesis was verified using VIGS method in the rose. In this study, we were able to show that lncRNAs may play important roles in floral scent production in the rose. This study also improves our understanding of how plants regulate their secondary metabolism by lncRNAs.

## Introduction

Approximately 90% of the eukaryote genome is transcribed (Wilhelm et al., 2008), but only 1-2% of the genome has a protein-coding capacity (Consortium, 2007), and the majority of the genome is transcribed as non-coding RNAs (ncRNAs). Small ncRNAs with length of less than 200 bp, such as microRNAs (miRNAs), small interfering RNAs (siRNAs), piwi-interacting RNAs (piRNAs), transacting siRNAs (ta-siRNAs), and natural antisense transcript siRNAs (NAT-siRNAs), have received considerable attention in the last decade for their essential roles in post-transcriptional and transcriptional regulation in eukaryotes (Simon and Meyers, 2011; Samad et al., 2017; Sun et al., 2019). Among them, the most well-known miRNAs are a class of RNAs with lengths of 20–24 bp that are highly conserved throughout evolution and regulate the growth and development of organisms by cleaving and degrading target gene transcripts or inhibiting translation through complementary pairing with the bases of target sites. In contrast, lncRNAs are typically larger than 200 bp but poorly conserved; they interact with large molecules, such as DNA, RNA, and proteins, and regulate protein modification, chromatin remodeling, protein functional activity, and RNA metabolism *in vivo* through cis- or trans-activation at the transcriptional, post-transcriptional, and epigenetic levels (Chekanova, 2015).

In the past decade, thousands of lncRNAs have been identified in plants, including *Arabidopsis thaliana* (Di et al., 2014; Wang et al., 2014; Zhu et al., 2014; Moison et al., 2021; Liu et al., 2022), *Medicago truncatula* (Wen et al., 2007), *Triticum aestivum* (Xin et al., 2011; Zhang et al., 2016; Lu et al., 2020), *Oryza sativa* (Shin et al., 2018; Yu et al., 2020; Zhang et al., 2020; Chen et al., 2021), *Zea mays* (Boerner and McGinnis, 2012; Li et al., 2014), *Manihot esculenta* Crantz (Li et al., 2022), *Solanum lycopersicum* (Zhu et al., 2015; Jiang et al., 2019), *Cuscuta* spp. (Wu et al., 2022), *Populus trichocarpa* (Shuai et al., 2014), *P. tomentosa* (Chen et al., 2015; Chen et al., 2022), and *P.×euramericana* (Wang et al., 2017). Although the regulatory mechanisms of lncRNAs have been elucidated widely, they are mostly derived from animals, and only a few lncRNA mechanisms in plants have been revealed, resulting in a lack of systematic and consensus lncRNA regulatory mechanisms in the plant (Wu et al., 2020). Two novel intergenic lncRNAs in tomato, lncRNA1459 and lncRNA1840, play a regulatory role in tomato fruit ripening (Zhu et al., 2015), while a lncRNA in rice, referred to as long-day-specific male-fertility-associated RNA (LDMAR), regulates photoperiod-sensitive male sterility (Ding et al., 2012). In rice and maize, there is an association of some lncRNAs and their polymorphisms with agricultural traits (Wang et al., 2015). Two lncRNAs—COOLAIR (cool-assisted intronic non-coding RNA) and COLDAIR (cold-assisted intronic non-coding RNA)—are found to regulate vernalization by negatively regulating a MADS-box transcription factor FLC that represses flowering in *Arabidopsis* (Heo and Sung, 2011; Sun et al., 2013). Some lncRNAs were found to be endogenous target mimics (eTMs) of miRNAs, indicating a new mechanism for regulating miRNA activity (Wu et al., 2013; Jiang et al., 2019). In *Arabidopsis thaliana*, lncRNA SABC1 recruited the polycomb repressive complex 2 to its neighboring gene *NAC3* to decrease its transcription *via* H3K27me3 (Liu et al., 2022). This evidence, highlighting the essential and varied functions of lncRNAs, demonstrates the importance of discovering and identifying lncRNAs in different biological processes and the need to elucidate their functional mechanisms.

Floral scent primarily attracts pollinators to angiosperms to facilitate in fertilization (Dudareva et al., 2004), but also functions in plant defense (Caruso and Parachnowitsch, 2016), brings mental pleasure to humans, and provides essential oils and flavors for the food and perfume industries (Grammer et al., 2003). Increasing numbers of flower volatile biosynthesis genes have been cloned but the complete regulatory mechanism(s) has yet to be elucidated (Muhlemann et al., 2014), and lncRNAs in floral scent synthesis remain predominantly unknown. Consequently, the identification and characterization of novel lncRNAs is crucial to understand the function of lncRNAs in floral scent.

Rose is one of the most commonly cultivated ornamental plants in the world, popular in gardens and as cut flowers, but are also important sources of essential oils for perfumes and cosmetics due to their floral scent (Magnard et al., 2015). However, in the process of rose breeding over hundreds of years, the focus on cut flowers and visual attributes has disadvantaged scent traits (Vainstein et al., 2001). Rose probably manufactures the most diverse scent compounds based on the emission of hundreds of volatile molecules. Any variation in the composition of the volatile molecules, in both quality and quantity, could lead to different rose scent profiles (Joichi et al., 2005; Bendahmane et al., 2013). Three major scent molecule classes were involved in roses: the terpenes, including rose oxide, geraniol, linalool, citronellol, nerol and so on; the benzenoids/phenylpropanoids, such as 2-phenylethanol (2-PE), 2-phenylethyl acetate, 3,5-dimethoxytoluene (DMT), 1,3,5-trimethoxybenzene (TMB) methyleugenol, methylisoeugenol and so on; the fatty acid derivatives, including *cis*-3-hexenyl-1-alcohol, 2-hexenyl acetate, *cis*-3-hexenyl acetate (Schnepp and Dudareva, 2008). Regulatory factors were revealed to promote the production of floral scent compounds more efficiently compared with synthetase genes, indicating a powerful tool to modify the floral scent trait (Zvi et al., 2012). However, although dozens of genes in synthesis pathway of rose floral scent have been identified and functionally validated in the past decade, there is limited information available on the regulatory mechanism in the rose (Shi and Zhang, 2022). Only one transcription factor (TF), RhMYB1 was found to probably play a role in rose floral scent production, but its function has not been validated (Yan et al., 2011). In rose petals, the miR156-*SPL9* regulatory hub is proposed to orchestrate the production of both colored anthocyanins and certain terpenes, by permitting the complexation of preexisting MYB-bHLH-WD40 proteins (Raymond et al., 2018).

This study used the rose cultivar ‘Tianmidemeng’ with a heavy floral scent to identify and analyze lncRNAs through strand-specific RNA-seq of petal samples from three flowering stages of the rose. Based on genome location and differential expressions of the lncRNAs, together with weighted gene co-expression network analysis (WGCNA), lncRNAs related to floral scent were identified. A total of 13,957 putative lncRNAs were discovered. Rose lncRNAs are shorter and harbor fewer exons and less coding potential compared with the protein-coding genes. Hundreds of lncRNAs showed significantly differential expression among the three flowering stages of the rose, and target prediction for lncRNAs coupled with WGCNA supported the role of these lncRNAs in floral scent production. Moreover, WGCNA further correlated the differentially expressed lncRNAs to individual floral scent compounds. Findings from the study suggest that lncRNAs are instrumental in the regulation of floral scent production and provide new insights into the study of floral scent.

## Materials and methods

### Plant material and growth conditions

**Figure 1:**
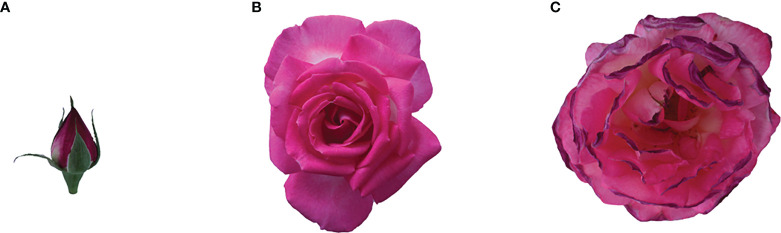
Flower developmental stages in *Rosa hybrida* ‘Tianmidemeng’. **(A)** Early-flowering (EF): sepals are unfolding, the petals are closed and become red, with almost no fragrance; **(B)** Semi-flowering (SF): the outer 2-3 layers of petals are unfolded, but the petals are still closed, the color is rose-red and the aroma is strong; **(C)** Late-flowering (LF): petals begin to wilt and the color begins to fade, with some fragrance.

The plant material ‘Tianmidemeng’ (*R. hybrida*) was planted in the natural environment of the campus of China Agricultural University in Haidian district, Beijing. Based on the open state of the flower, we divided the flower development into three stages: 1) early-flowering (EF), of which the sepals are slightly unfolded while the petals are still closed, the petals are becoming red and have little fragrance; 2) semi-flowering (SF), of which the outer 2-3 layers of petals are unfolded while the inner part are still closed, the petal color is rose-red, and the fragrance is rich; 3) late-flowering (LF), of which the petals are all unfolded but begin to wilt, the petal color begins to fade, and some fragrance still remains (**Figure 1A–C**). All fresh petal samples from development stages were collected at 9:00 a.m. The flower materials collected for each sample were divided in half: one part was used for gas chromatography-mass spectrometry (GC-MS) analysis, and the other part was for RNA-seq analysis after immediately freezing in liquid nitrogen. For every sample, three replicates were prepared.

### Floral scent collection and GC-MS analysis

For each sample, 3 g petals were quickly placed into a 100-mL sample vial, and 10 μL ethyl caprate (0.865 μg·μL^-1^; Sigma Ltd. Co., New York, USA) was subsequently added as the internal standard. The vial was then sealed rapidly with a rubber septa. For extracting and concentrating the floral volatiles in the vial, a solid-phase microextraction (SPME) manual headspace sampler was used with a 100-μm polydimethylsiloxane (PDMS) fiber embedded in it (Supelco, Bellefonte, PA, USA). The extraction and concentration were lasted for 40 min at 30°C.

GC-MS was carried out using a Trace DSQ-GC-MS (Thermo Corporation, Waltham, MA, USA). The flow rate of the helium carrier gas in the DB-5MS fused-silica capillary column (30 m × 0.25 mm × 0.25 mm film) was 1.00 mL·min^-1^. Then, the sample was injected into the injector port at the temperature of 200°C. The column temperature was programmed as follows: the initial temperature was set at 50°C for 1 min, and then increased to 200°C at a rate of 5°C·min^-1^, finally increased to 230°C at 8 °C·min^-1^ and maintained for 8 min. The volatile compounds were identified by matching the resulting mass spectra with the NIST 11 library (National Institute of Standards and Technology, Gaithersburg, MD, USA), retention index and relative reports from the literature. Quantitative analysis was carried out by comparing peak areas of volatile compounds with that of the internal standard (Feng et al., 2014). The mass fraction was calculated as compound emission rate (μg·g^-1^·h^-1^) = {peak area of compound/peak area of internal standard × concentration of internal standard (μg·μL^-1^) × volume of internal standard}/sample mass (g)/extraction time (h).

### RNA extraction and pair-end strand-specific RNA sequencing

The total RNA of each sample was extracted using a universal RNA extraction kit (Tiangen Biotech Co., Ltd., Beijing, China) according to the manufacturer’s instructions. RNA concentration and quality were determined with a Qubit 2.0 fluorometer (Life Technologies, Carlsbad, CA, USA), and a spectrophotometer (NanoPhotometer; Implen, Calabasas, CA, USA), respectively. RNA integrity was measured using a Bioanalyzer 2100 system with the RNA 6000 Nano Assay kit (Agilent, Carlsbad, CA, USA).

Nine strand-specific RNA libraries were prepared with an insert size of ~250–500 nucleotides using a UTP method (Parkhomchuk et al., 2009), and then were sequenced by Biomarker Technologies Corporation (BMK, Beijing, China) on the Illumina HiSeq 2000 platform with the 150-bp paired-end method and a sequencing depth of ~53 million reads per library (**Table 1**).

**Table 1:** Statistics of transcriptome data of *Rosa hybrida* ‘Tianmidemeng’.

Samples	Raw reads	Clean reads	Clean base pairs (Gb)	Q20 (%)	Q30 (%)
EF1	49,541,392	49,198,496	7.35	96.65	91.91
EF2	52,062,754	51,724,058	7.73	96.61	91.82
EF3	46,408,334	46,117,440	6.9	96.8	92.21
SF1	57,575,304	57,192,114	8.55	96.62	91.79
SF2	49,469,348	49,154,698	7.35	96.71	91.98
SF3	51,119,628	50,781,356	7.59	96.65	91.88
LF1	53,826,078	53,481,560	8.01	96.61	91.83
LF2	51,315,158	50,964,450	7.62	96.75	92.16
LF3	67,557,308	67,102,068	10.02	96.77	92.2

### Assembly of RNA transcripts

Barcode and adaptor sequences were removed from the sequencing reads by the quality checking and trimming processes. Any rRNA sequences were eliminated by aligning all reads to plant rRNA sequences using the Short Oligonucleotide Analysis Package (SOAP2; http://soap.genomics.org.cn/soapaligner.html). The clean reads from each library were then aligned with the reference genome of the rose ‘Old blush’ (ftp://ftp.ncbi.nlm.nih.gov/genomes/all/GCF/002/994/745/GCF_002994745.1_RchiOBHm-V2/GCF_002994745.1_RchiOBHm-V2_genomic.fna.gz) using Hisat2 (version 2.1.0; https://ccb.jhu.edu/software/hisat2/index.shtml). The alignments were used to assemble transcripts using StringTie (version v1.3.6, http://ccb.jhu.edu/software/stringtie/).

### Bioinformatics analysis for identification of lncRNAs

The assembled transcripts from each library were merged by Cuffmerge to remove those with uncertain directions or those shorter than 200 nt. Cuffcompare was then used to compare transcripts with the rose genome annotated protein sequences (ftp://ftp.ncbi.nlm.nih.gov/genomes/all/GCF/002/994/745/GCF_002994745.1_RchiOBHm-V2/GCF_002994745.1_RchiOBHm-V2_protein.faa.gz). The non-redundant transcripts exhibiting significant alignment (*P*<1.0E-10, identity >90%, coverage >80%) with rose proteins were excluded. According to rose genome annotation, all resulting transcripts that aligned to housekeeping ncRNAs (including rRNAs, tRNAs, snRNAs, and snoRNAs) were also removed.

Transcripts with short ORFs (<100 amino acids) were detected for the open reading frame (ORF) filter. The longest consecutive codon chain was defined as the putative ORF of the lncRNA candidate. In addition, transcripts were aligned to the protein family (Pfam) database using the HMMER 3.0 program (profile hidden Markov model software) (Finn et al., 2011) with an E-value threshold of 10^−5^ to filter transcripts containing a known protein domain. The resulting transcripts were tested for protein-coding potential using the Coding Potential Calculator (CPC) software, and only transcripts with a CPC score of <0 were retained and considered as lncRNAs (Kong et al., 2007). The intersection of transcripts with no coding potential in the results of the two software analyses were considered as rose lncRNAs.

### Classification of lncRNAs

Based on location relative to the nearest protein-coding genes, the annotated lncRNAs were subdivided into four categories: (i) antisense lncRNAs, which overlap with exons of a protein-coding transcript on the opposite strand; (ii) lncRNAs without any overlap with other protein-coding genes are classified as intergenic lncRNAs (lincRNAs); (iii) lncRNAs with some overlap with genes on the same strand are classified as sense overlapping lncRNAs; and (iv) lncRNAs in some protein-coding loci but without any overlap with exons of protein-coding genes are classified as sense intronic lncRNAs (Harrow et al., 2012).

## Distribution of transcript length, exon number, and ORF length of lncRNAs and protein-coding

### Genes in rose

LncRNAs and protein-coding genes were analyzed for transcript length and exon number as followings (Zhu et al., 2015). Transcript length categories were <300, 300–400, 400–500, 500–600, 600–700, 700–800, 800–900, 900–1000, and >1000 nucleotides. Exon number categories were: 1, 2, 3, 4, 5, 6, 7, 8, 9, 10, and >10. The proportions of different kinds of lncRNAs and protein-coding transcripts were then calculated.

### Target gene prediction of lncRNAs in rose

There are two predominant mechanisms by which lncRNAs regulate target genes (Schmitt and Chang, 2016). Co-location means that a lncRNA may regulate the adjacent protein-coding genes, while co-expression means that a lncRNA regulates downstream genes through correlated expression. The threshold for the co-location mechanism was set to 100 kb upstream or downstream of the lncRNA location in the chromosome. For co-expression prediction, the pearsonr function was called by the python statistics module of scipy.stats to calculate pearson correlation coefficients of expression levels between lncRNAs and mRNAs in the trans-loci. It was conducted only when the sample number was bigger than five and the threshold for the pearson correlation coefficient was set to greater than 0.95 (Kopp and Mendell, 2018; Bao et al., 2019).

### Differential expression of LncRNAs and mRNAs among developmental stages of rose flower

Using the cuffdiff program, both differentially expressed lncRNAs and mRNAs among flower developmental stages were identified (Trapnell et al., 2012). LncRNAs and mRNAs exhibiting |log_2_ (fold change)| ≥1 and adjusted *P*-values <0.05 were selected as differentially expressed.

### Co-expression network analysis

Key lncRNAs correlated to flower volatiles were identified based on dynamic lncRNA expression changes in tissues of different flowers using the R package WGCNA (Langfelder and Horvath, 2008). For the sample number—including the biological replicates—needed by WGCNA was at least 15, the GC-MS and RNA-seq data of our another three rose cultivars’ flowers were recruited. The three cultivars—’Elle’, ‘First blush’ and ‘Qingge’—were parents and sister of ‘Tianmidemeng’ and possessed distinctive floral scent profiles, respectively. The GC-MS data was obtained from their flowers in the same condition and method as ‘Tianmidemeng’, while the RNA-seq data was obtained from their flowers in the same condition but with non-strand-specific RNA sequencing method. LncRNAs were isolated from RNA-seq data of the three cultivars and their expression levels were calculated with the same methods as ‘Tianmidemeng’. Parameters were set up as power = 6, minModuleSize = 6, deepSplit = 4, mergeCutHeight = 0.1, and MEDissThres = 0.15. The TO value (topological overlap, unsigned) was calculated for each pair of lncRNAs (Ravasz et al., 2002; Li and Horvath, 2006; Yip and Horvath, 2007) and a lncRNA cluster tree was subsequently constructed by hierarchical clustering method and further split into modules by the method of dynamic treecut (Langfelder and Horvath, 2008). Eigengenes (ME) of each module were evaluated by principal component analysis (PCA). To correlate flower volatiles with modules, the contents of flower volatiles in every tissue in the developmental process were listed and assembled into a matrix. The coefficient factors between the matrix and MEs were calculated. For each flower volatile, modules with top three coefficient factors were selected.

### Quantitative reverse transcription-PCR

Validation of the RNA-seq results was conducted by qRT-PCR analysis to ten potential floral-scent-related lncRNAs. RNA samples for the three flowering stages were isolated from the same flower tissues as RNA-seq libraries, respectively. The cDNA for each sample was then synthesized using ReverTra Ace qPCR RT Master Mix (Toyobo, Japan). Primers for qRT-PCR were designed using Primer Premier software (version 5.0), listed in **Supplementary Table 1**. A StepOnePlusTM Real-Time PCR System (Life Technologies, Carlsbad, CA, USA) was used to detect relative lncRNA expression levels with the SYBR^®^ Green Real-Time PCR Master Mix (Toyobo, Japan). Three biological replicates were performed, and the reactions were performed in triplicate for each run. The quantification of the relative expression of the genes at different times was performed using the delta-delta Ct method as described by Livak and Schmittgen (Livak and Schmittgen, 2001). All data were expressed as means ± standard deviation (SD) after normalization. *GAPDH* was used as an internal control. Linear regression analysis was conducted using the fold-change values of qRT-PCR and RNA-Seq.

### Functional examination of rose lncRNAs

Virus-induced gene silencing (VIGS) was used to examine the functions of the candidate lncRNAs for floral scent production in the rose. LncRNA fragments of 300-500bp were amplified from the cDNA of ‘Tianmidemeng’, and then inserted into the vector of pTRV2 with a homologous recombination method The pTRV1, pTRV2 and pTRV2-lncRNA constructs were transformed into the competent cells of *Agrobacterium* strain GV3101, respectively. Monoclonal colonies of GV3101 with pTRV1, pTRV2 or pTRV2-lncRNA vectors were cultured in LB medium (pH 5.6) containing 10 mM MES and 20 μM acetosyringone with kanamycin, gentamycin, and rifampicin antibiotics at 28 °C for 24 h, The cultures were collected by centrifugation and then resuspended in the infiltration buffer (10 mM MgCl2, 200 μM acetosyringone, 5% sucrose) until the OD600 of the resuspension buffer arrived to 1.2-1.5. The infection buffer was prepared by mixing the resuspension buffers of pTRV1 and pTRV2, or pTRV2-lncRNA at a ratio of 1:1, and then placed in the dark for 2-3 h.

Flowers of rose cultivar ‘Tineke’ in the EF stage were pricked by a needle in four directions, and then submerged in the infection buffer and subjected to a vacuum at 0.8-1 bar twice, each for 60 s. Infiltrated flowers were washed with distilled water and then grown in clean water at 8 °C in dark for 3 d and then in the greenhouse for another 3 d. Flowers infiltrated by infection buffer with pTRV1 and pTRV2 were set as controls. Each infiltration was carried out with 10 biological replicates. The collection and measurement of samples for GC-MS and qRT-PCR were conducted as mentioned above. Primers for VIGS and qRT-PCR were designed using Primer Premier software (version 5.0), listed in **Supplementary Table 1**.

## Results

### Floral scent changes of rose ‘Tianmidemeng’ during three flowering stages

The compositions and contents of floral volatiles were detected by GC-MS for three flowering stages of the rose ‘Tianmidemeng’, respectively (**Figure 1**). The non-floral-scent components, such as aliphatic hydrocarbons, alkenes, alcohols, aldehydes, acids, and esters, were excluded according to the criteria for the three classes of floral scent compounds and relevant reports about rose floral scent (Joichi et al., 2005; Schnepp and Dudareva, 2008; Bendahmane et al., 2013). Then the floral scent components in three flowering stages were obtained, of which the numbers were 16, 36, and 44, respectively, including terpenoids, phenylpropanoids/benzenoids, and fatty acid derivatives (**Supplementary Table 2**).

**Figure 2:**
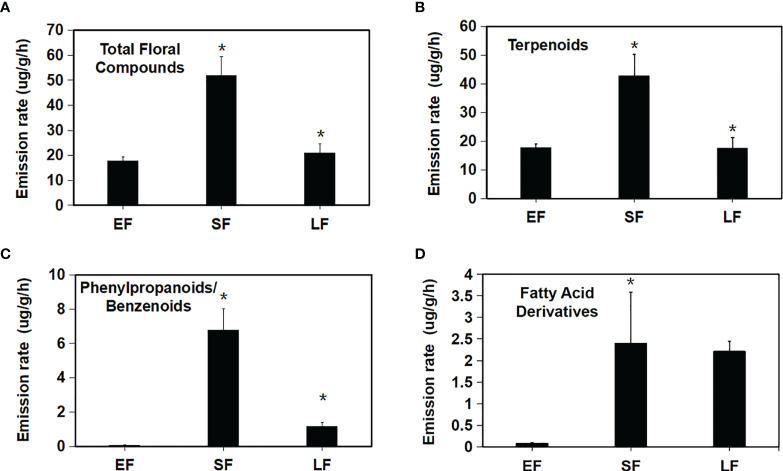
Variation in compound emission rates across flower developmental stages in *Rosa hybrida* ‘Tianmidemeng’. **(A)** Emission variation of total compounds across flower developmental stages; **(B)** Emission variation of floral terpenoids across flower developmental stages; **(C)** Emission variation of floral phenylpropanoids/benzenoids across flower developmental stages; **(D)** Emission variation of floral fatty acid derivatives across flower developmental stages. Emission rates during SF were the greatest for nearly all floral compounds. Means with asterisk (*) are significantly different (Student’s *t* test, P < 0.05).

Based on the internal standard, the release amount (release rate) of each floral scent component in the three flowering periods was calculated (**Figures 2A–D**). Total release in the initial stage was 4.478 ug·g^-1^·h^-1^, and this increased significantly to 51.84 ug·g^-1^·h^-1^ in the semi-flowering stage but was 20.93 ug·g^-1^·h^-1^ in the final stage, which was a significant decrease compared with that in the semi-flowering stage (**Figure 2A**). This showed that floral scent synthesis of ‘Tianmidemeng’ changed significantly following the development of flower and reached the highest value in the semi-flowering stage. When floral scent components were classified into the three classes, it was found that the terpenoids accounted for the majority of the floral scent in the three flowering periods: 98.9% at the bud stage, 82.3% at the semi-flowering stage, and 83.8% at the late-flowering stage, and the changes of release amount were consistent with those of the overall floral scent (**Figure 2B**). The release changes of phenylpropanoids/benzenoids were the same as for the terpenoids (**Figure 2C**), while the release changes of fatty acid derivatives were similar to those of terpenoids and phenylpropanoids/benzenoids but the decrease was not significant from semi-flowering to late-flowering stages (**Figure 2D**). These results demonstrated that the synthesis of fatty acid derivatives was less affected by petal senescence compared with the other two classes of compounds (terpenoids and phenylpropanoids/benzenoids).

In summary, floral scent synthesis of the rose ‘Tianmidemeng’ was regulated by the flowering process, and the semi-flowering stage was the period with the highest synthesis of various floral scent components. However, the responses of three kinds of floral scent compounds to the flowering process were slightly different; terpenoids and phenylpropanoids/benzenoids were sensitive to flower opening and aging, while the fatty acid derivatives were less sensitive to flower aging.

### Identification of lncRNAs in rose flowers

**Figure 3:**
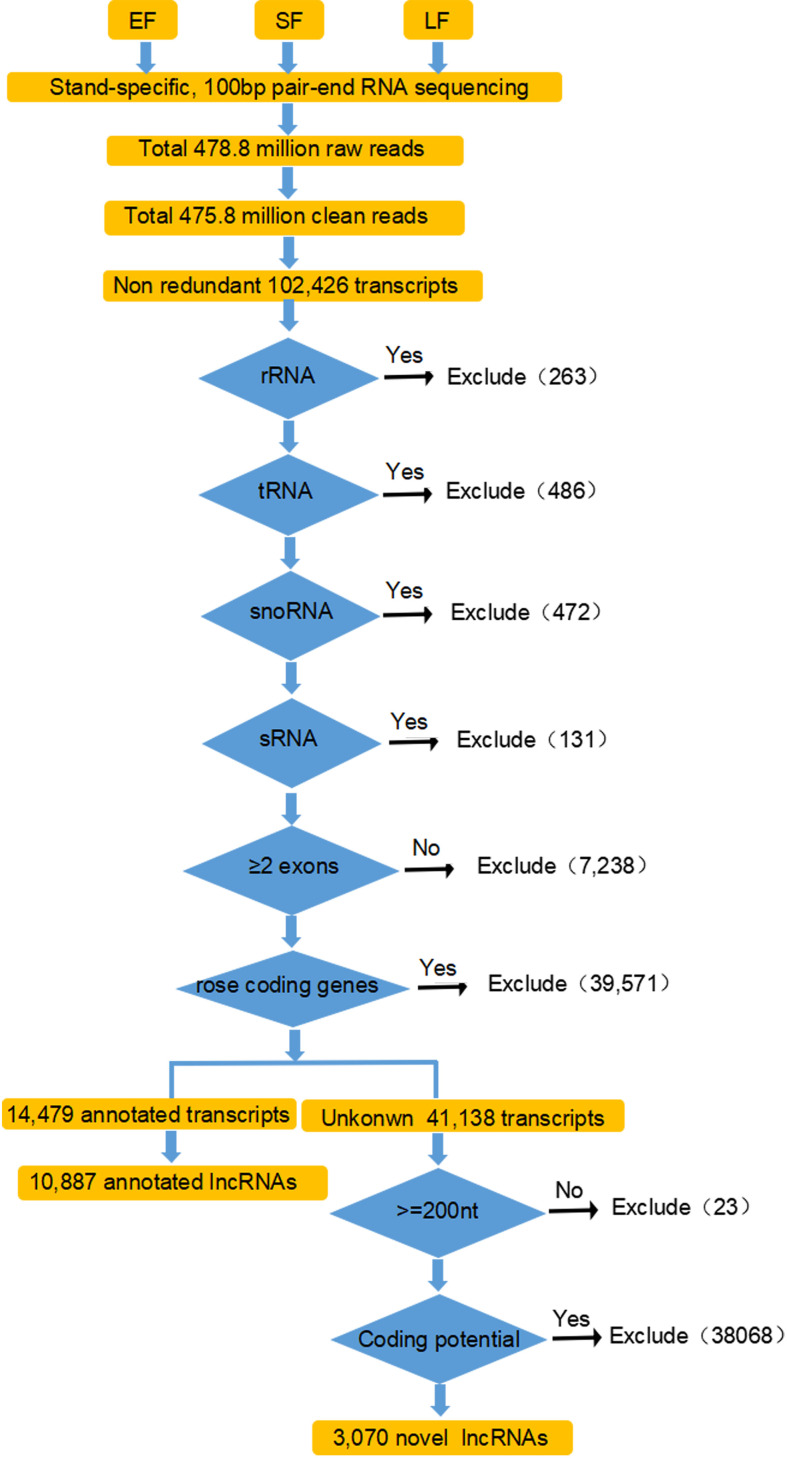
Detailed flow schematic for identification of rose lncRNAs. Paired-end strand-specific RNA-Seq was performed for rose flowers at the EF, SF and LF stages. Clean reads were mapped and assembled according to the known rose genome using Hisat2 and StringTie. Transcripts were filtered with the five criteria for the identification of putative lncRNAs. (i) not housekeeping ncRNAs; (ii) number >2 exons; (iii) not rose coding genes; (iv) length >200 nucleotides and ORF <100 amino acids; and (v) not encoding known protein domains and little coding potential. At each step, a blue arrow indicates those transcripts which were passed by the filter; a black arrow, those that were excluded. The number of transcripts that did not pass the filter is shown.

To identify lncRNAs in rose flowers, paired-end ssRNA-Seq for early-flowering, semi-flowering, and late-flowering stages of ‘Tianmidemeng’ was performed in three biological replicates. A total of ~476 million clean reads were obtained (**Table 1**; **Figure 3**), and 102,426 unique transcripts were assembled (**Figure 3**).

To distinguish lncRNAs, five sequential stringent filters were applied to the 102,426 transcripts (**Figure 3**). First, the transcripts were filtered with rose ncRNAs. A total of 263 rRNAs, 486 tRNAs, 472 snoRNAs, and 131 sRNAs were excluded according to the ‘Old blush’ genome annotation, leaving 101,074 transcripts. Among these, transcripts with a single exon were filtered for low reliability, and 95,188 transcripts with an exon number ≥2 were selected (**Figure 3**). The resulting transcripts were then filtered with rose coding gene sequences. Almost 42% (39,571) of transcripts were coding genes, and the remaining 58% (55,617) might potentially be non-coding transcripts, consistent with other studies and showing that ncRNAs were widely transcribed (**Figure 3**) (Heo et al., 2013).

Among the 55,617 potential non-coding transcripts, 14,479 were annotated by the ‘Old blush’ genome annotation, with 10,887 annotated as lncRNAs (**Figure 3**). The unannotated 41,138 transcripts were further analyzed for novel lncRNAs. Two criteria—longer than 200 nucleotides and unable to encode polypeptides longer than 100 amino acids—were applied to the 41,138 transcripts, and 41,115 transcripts were recovered (Li et al., 2014; Shuai et al., 2014) (**Figure 3**). Coding potential is the key condition to judge whether a transcript is lncRNA. Transcripts with potential protein-coding domains were therefore further filtered by comparison with the Pfam database. Finally, after the assessment by CPC software, transcripts without protein-coding potential were obtained as the novel lncRNAs. After employing three stringent criteria, 3070 transcripts were considered as novel lncRNAs. Thus, a total set of 13,957 transcripts were obtained and defined as rose lncRNAs, including 10,887 annotated lncRNAs and 3070 novel lncRNAs (**Supplementary Table 3**).

### Computational classification and characteristics of rose lncRNAs

**Figure 4:**
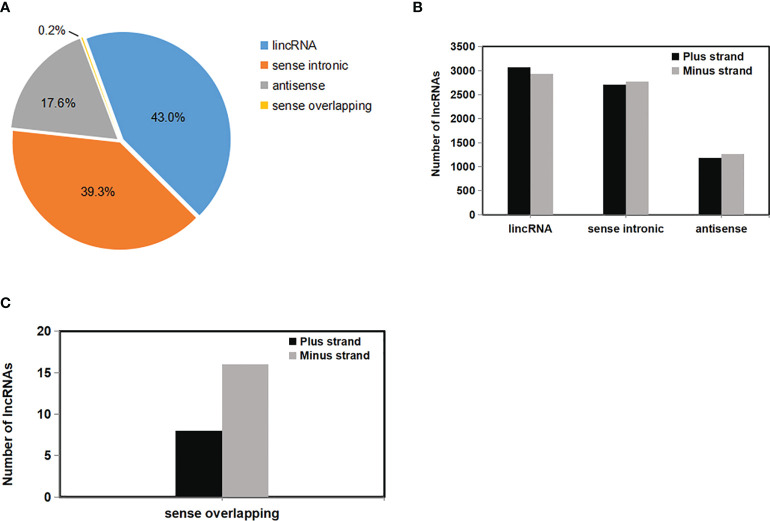
Annotation classification of 13,957 rose lncRNAs. **(A)** Classification of rose lncRNAs according to their genomic position and overlap with protein-coding genes. **(B)** Numbers of lncRNAs in the Watson or Crick strand for each of the three main classes were labelled on the columns (intergenic, intragenic, and antisense lncRNAs). **(C)** Number of lncRNAs in the Watson or Crick strand for sense overlapping class was labelled on the columns. The proportion of the four kinds of lncRNAs was calculated.

LncRNAs were further classified into four types according to the location relative to the nearest protein-coding genes. These types are: intergenic (lincRNA), sense intronic, sense overlapping, and antisense lncRNAs (**Figure 4A**) (Harrow et al., 2012). Most of the lncRNAs—5999 lncRNAs (43.0%)—were located in intergenic regions, whereas 2453 (17.6%) and 24 (0.2%) of the lncRNAs were either antisense of or overlapped with protein-coding genes (**Figure 4A**). This observation was consistent with previous studies (Li et al., 2014). In addition, 5481 lncRNAs (39.3%) were transcribed from inside genes (most from introns), which was similar to the result obtained for *Arabidopsis* but widely divergent to the result in tomato (Wang et al., 2014; Zhu et al., 2015). The numbers of three types of lncRNAs—lincRNA, sense intronic, and sense overlapping—from plus and minus strands (Watson and Crick strands) were similar (**Figure 4B**), while the number of sense overlapping lncRNAs was different from these (**Figure 4C**).

**Figure 5:**
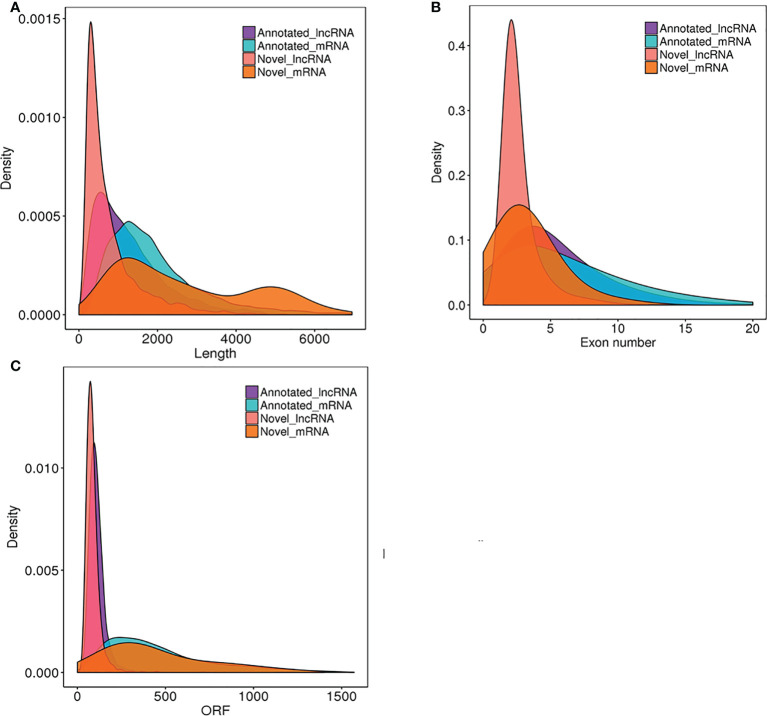
Comparison of the length, the exon number and ORF between lncRNAs and protein-coding transcripts. The distribution of length **(A)**, numbers of exons **(B)** and ORF of identified lncRNAs **(C)** in comparison with all protein-coding transcripts of the rose ‘Old Blush’.

Plant lncRNAs are reported to be shorter and harbor fewer exons compared with protein-coding genes (Li et al., 2014; Shuai et al., 2014). To determine whether rose lncRNAs shared these features, all the 42,767 genes predicted in the genome of the rose ‘Old Blush’ were applied to analyze the distribution of length and exon number of the 13,957 lncRNAs. **Figure 5A** shows that ~55% of the lncRNAs ranged in size from 200 to 1000 nucleotides, with only 45% comprising >1000 nucleotides. In contrast, for the protein-coding transcripts, ~80% comprised >1000 nucleotides. Most (70%) of the genes encoding rose lncRNAs only contained ≤5 exons, while the number of exons for the protein-coding genes ranged from one to ≥20 (**Figure 5B**). All rose lncRNAs possessed ORFs with a length shorter than 100 amino acids, while the ORF lengths of protein-coding genes ranged from one to ≥1000 AA (**Figure 5C**). Collectively, these results indicated that most of the rose lncRNAs are relatively short and contain only a few exons compared to protein-coding genes.

### Identification of floral-scent-related lncRNAs

**Figure 6:**
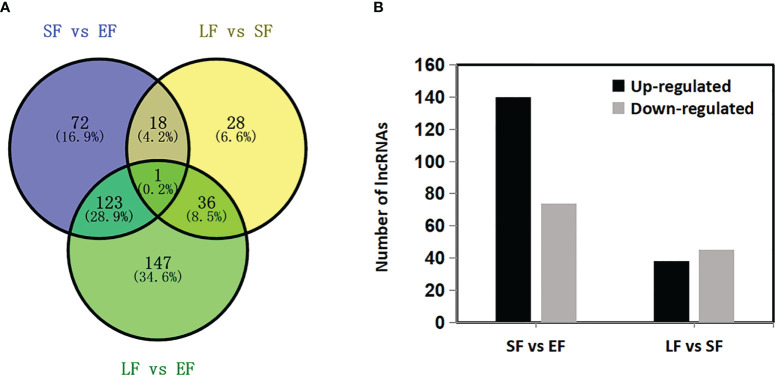
Differentially expressed lncRNAs in flower developmental stages in rose ‘Tianmidemeng’. **(A)** Venn diagram of differentially expressed lncRNAs when comparing successive flower developmental stages from early flowering (EF), semi-flowering (SF), to late flowering (LF). **(B)** Graphic presentation of up- and down-regulated differentially expressed lncRNAs when comparing successive flower developmental stages.

The emission rate of floral-scent compounds changed among the flowering stages of rose. Therefore, it was hypothesized that floral-scent-related mRNAs and lncRNAs might be present in roses. A total of 9664 mRNAs were differentially expressed among the three flower-development stages of the rose ‘Tianmidemeng’ (**Supplementary Table 4**). For lncRNAs, 534 of them were identified as differentially expressed. Among them, 109 lncRNAs were excluded as their expression levels were lower than 0.5 in all three stages, leaving 425 differentially expressed lncRNAs in the research (**Supplementary Table 5**). From the early-flowering stage to the semi-flowering stage, 214 lncRNAs were differentially expressed, of which 140 and 74 were upregulated and downregulated, respectively. From the semi-flowering stage to the late-flowering stage, 83 lncRNAs were differentially expressed, of which 38 and 45 were upregulated and downregulated, respectively (**Figures 6A, B**). There were 19 lncRNAs expressed in both of these processes and these were deemed core candidate lncRNAs. Among the core lncRNAs, the expression levels of nine lncRNAs increased from bud to semi-flowering stage and then subsequently decreased, while nine lncRNAs decreased first and then increased (**Figure 6A**). This indicated that the nine and nine lncRNAs might have positive and negative roles, respectively, in floral scent synthesis.

LncRNA is rich in biological functions and is involved in various important physiological processes. LncRNA can regulate the expression of target genes at transcriptional and post transcriptional levels (Schmitt and Chang, 2016). The two predominant mechanisms by which lncRNA regulates target gene are co-location, where the lncRNA may regulate the adjacent protein-coding genes, and co-expression, where the lncRNA regulates downstream genes through correlated expression. On the basis of these two mechanisms, 10,075 of the total 13,957 lncRNAs were predicted to possess a set of 29,622 target genes (**Supplementary Table 6**).

The above correlation prediction was also used to search for lncRNAs related to floral scent production. First, all genes involved in downstream synthesis pathways of floral scent compounds from were identified from the rose mRNAs in this research, and a total of 54 genes were obtained. Using the correlation prediction between lncRNA and target genes, a total of 849 corresponding lncRNAs were identified for the 54 genes, among which, 141 corresponding lncRNAs were differentially expressed (**Supplementary Table 7**). Among the 141 lncRNAs, five were core lncRNAs, including TCONS_00007202, TCONS_00008447, TCONS_00117855, XR_002924185.1, and XR_002931444.1.

The expression changes of upstream genes in floral scent synthesis pathways were usually irregular, hence their corresponding lncRNAs with irregular expression changes might also be candidates for floral scent. Accordingly, 15 upstream genes were selected and 87 differentially expressed lncRNAs corresponding to these genes were identified (**Supplementary Table 8**). Among the 87 lncRNAs, three were core lncRNAs, and 84 were other potential lncRNAs. The three core lncRNAs—TCONS_00007202, TCONS_00008447, and TCONS_00117855—were also obtained in the analysis of downstream genes.

To summarize, a total of 103 candidate lncRNAs for floral scent production were identified. Among them, the core 19 lncRNAs were identified according to gene expression changes during the flowering process, including five that were further validated by correlation analysis between lncRNAs and target genes of downstream syntheses in the floral scent synthesis pathway, and the other 84 lncRNAs were identified according to correlation analysis between lncRNAs and target genes of upstream syntheses in floral scent synthesis pathway. These lncRNAs are likely to be involved in floral scent production in rose.

**Figure 7:**
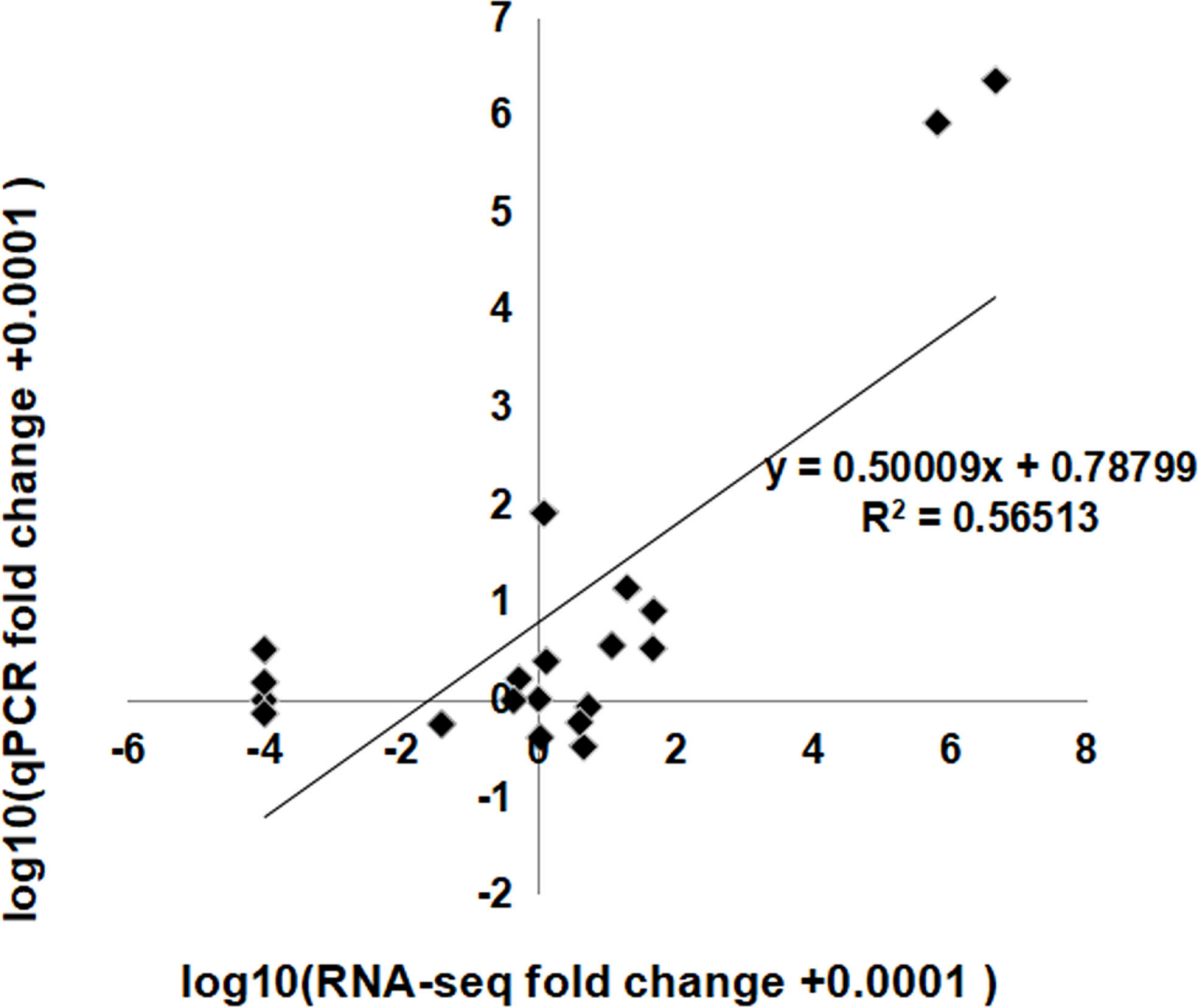
Coefficient analysis between lncRNA expression ratios obtained by RNA-seq and qRT-PCR data. RNA-Seq fold change refers to the ratios of RPKM values of SF, LF to EF for selected transcripts, while qRT-PCR fold change is the relative quantity of SF, LF normalized to the expression level of EF. EF, early-flowering stage; SF, semi-flowering stage; LF, late-flowering stage.

The expression profiles of some identified lncRNAs were confirmed by qRT-PCR. We randomly selected 10 lncRNAs—five with an up-down change and five with a down-up change—among the three flowering stages in sequencing results to conduct qRT–PCR validations (**Supplementary Figure 1**). The fold changes in the lncRNA expression levels measured by qRT–PCR were closely correlated to that by RNA-Seq (R^2 ^= 0.57, P<0.001) (**Figure 7**), showing a good consistency between the qRT-PCR and RNA-seq results. It further suggested that the lncRNAs would have a role in floral scent production.

### Identification of floral-scent-related TFs and their correlated lncRNAs

R2R3-MYBs and other TFs were reported to regulate floral scent synthesis in various plants (Muhlemann et al., 2014; Yeon and Kim, 2021). Based on previous publications about TFs for floral scent compounds, the sequences of all the TFs were collected—a total of 169 TFs, namely 139 MYBs, 7 bHLHs, 6 AP2/ERF, 4 WRKY, 5 NACs, 5 bZIPs, and 2 zinc finger-like, 1 ETHYLENE-INSENSITIVE3-like TFs—and used to screen for rose homolog transcripts. Among these TFs, 24 were differentially expressed, including 1 NAC, 18 MYBs, 2 ERFs, 1 bHLH, and 2 bZIPs, and all of them were potential candidate TFs for regulation of floral scent synthesis. Using the correlation prediction between lncRNAs and target genes, the 24 TFs were possible target genes of 208 lncRNAs. Among these 208 lncRNAs, 61 were differentially expressed and deemed as potential candidates (**Supplementary Table 9**). For example, for PbbHLH4, a TF regulating floral scent production in *Phalaenopsis*, the homolog in rose was *R. chinensis* ICE1-like transcription factor gene (ID: 112175393), and the correlated lncRNA was TCONS_00111355, which was among the core lncRNAs. The results of this analysis indicated that these lncRNAs are likely to be involved in floral scent production *via* some TFs in rose.

### WGCNA of differentially expressed lncRNAs

**Figure 8:**
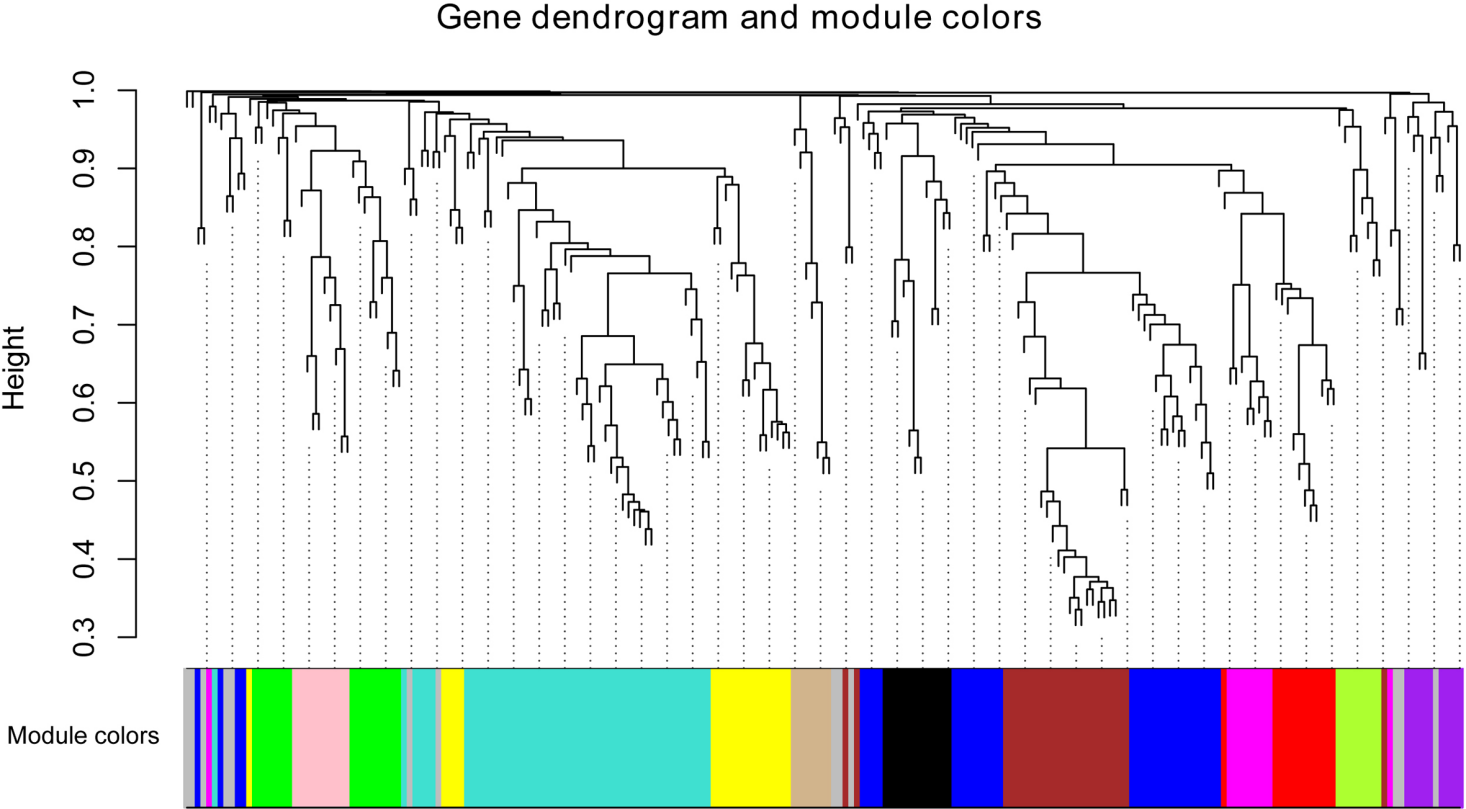
The co-expression network between lncRNAs and floral scent compounds. Clustering dendrogram of lncRNA and floral scent compounds, with dissimilarity based on the topological overlap, together with assigned module colors. Module colors are assigned according to module size, and the color gray is reserved for non-module lncRNAs.

WGCNA was employed to correlate lncRNAs with individual floral volatile compounds. As RNA-seq datas of the other three cultivars were obtained using non-strand-specific RNA sequencing method, less lncRNAs were identified from them compared to ‘Tianmidemeng’. Finally, 224 of the 425 differentially expressed lncRNAs in ‘Tianmidemeng’ were identified from the other three RNA-seq datas and clustered into 13 modules by WGCNA (**Figure 8**). All lncRNAs in the modules are listed in **Supplementary Table 10**. A correlation map between modules and compounds was generated (**Supplementary Figure 2**) and the top three modules for each compound were selected according to the correlation rate.

By this method, 11 modules were identified as related to 19 floral volatiles of the rose, including black, blue, green, greenyellow, magenta, pink, purple, red, tan, turquoise, and yellow (**Table 2**). All the 11 modules were correlated with the 14 compounds of terpenoids, of which the green yellow module was most correlated to half of the terpenoid compounds, including geraniol, nerol, geranyl acetate, citronellyl_acetate, citral, β-Pinene, and dihydro-β-ionol, and secondly correlated to neral and neranyl acetate. Among the left 5 terpenoid compounds, trans-β-ocimene, 7,8-dihydro-β-ionone, and trans-β-ionone were most correlated to magenta module, while the most correlated module of β-copaene and aromandendrene was turquoise. Among phenylpropanoids/benzenoids, phenethyl alcohol, phenethyl acetate and DMT were all most correlated with lncRNAs in modules of purple. Another benzenoids methyleugenol was correlated with lncRNAs in modules of greenyellow, pink and green, while the fatty acid derivative 4-hexen-1-ol-acetate was correlated with modules of greenyellow, magenta and black.

**Table 2:** Modules identified by weighted gene co-expression network analysis (WGCNA) responsible for flower volatiles in rose ‘Tianmidemeng’.

Flower volatiles	Module 1	Module 2	Module 3
Geraniol	greenyellow	pink	green
Nerol	greenyellow	green	pink
Neral	purple	greenyellow	tan
Geranyl acetate	greenyellow	green	black
Neryl acetate	green	greenyellow	black
Citronellyl acetate	greenyellow	black	green
Citral	greenyellow	black	
β-Pinene	greenyellow	tan	pink
β-Copaene	turquoise	yellow	tan
Trans-β-Ocimene	magenta	red	blue
7,8-Dihydro-β-ionone	magenta	red	blue
Dihydro-β-Ionol	greenyellow	black	purple
trans-β-Ionone	magenta	red	blue
Aromandendrene	turquoise	yellow	green
Phenethyl alcohol	purple	tan	greenyellow
Phenethyl acetate	purple	pink	tan
DMT	purple	tan	greenyellow
Methyleugenol	greenyellow	pink	green
4-Hexen-1-ol, acetate	greenyellow	magenta	black

For every volatile, three modules with top high coefficient factors from all the 13 modules were selected, respectively.

A total of 11 core lncRNAs were involved in six modules, of which five were related to floral scent production according to the above analysis, including green, greenyellow, purple, tan, and turquoise. The lncRNAs TCONS_00007202, XR_002924185.1 and XR_002931444.1, predicted to regulate floral scent synthase genes in the above correlation analysis, were involved in tan and greenyellow modules, respectively. Furthermore, as a potential regulator of the TF *PbbHLH4* homolog for the synthesis of monoterpenes in the rose (ID: 112175393), the lncRNA TCONS_00111355 was involved in the green module, which was predicted as related to terpenoids production. The WGCNA results were consistent with the above correlation analysis and further validated the role of core lncRNAs in regulating floral scent production directly or *via* TFs.

### Silencing of one candidate lncRNA TCONS_00008447 changed emission of rose floral scent compound

**Figure 9:**
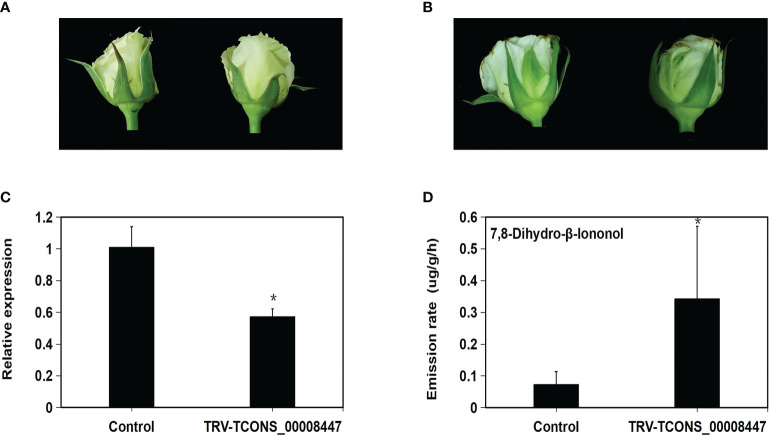
Silencing of lncRNA TCONS_00008447 changed the emission of floral scent compound. After 6 days, flowers of rose ‘Tineke’ infiltrated with TRV-TCONS_00008447 **(B)** showed a little unfolded and withered compared to TRV control flowers **(A)**. **(C)** qRT–PCR analysis of TCONS_00008447 transcript in TRV control and TRV-TCONS_00008447 flowers. *GAPDH* expression values were used for internal reference. **(D)** Emission variation of 7,8-dihydro-β-ionone in TRV-TCONS_00008447 flowers compared to TRV control. Error bars indicate ± SD of three biological replicates. Asterisks indicate a significant difference as determined by Student’s *t*-test (*P<0.05).

One core lncRNA TCONS_00008447 was selected to be silenced in rose ‘Tineke’ using the VIGS method. Its expression pattern presented an up-down change and was validated by the qRT-PCR (**Supplementary Figure 1**). After six days, flowers of rose ‘Tineke’ infiltrated with TRV-TCONS_00008447 (**Figure 9B**) showed a little unfolded and withered compared to TRV control flowers (**Figure 9A**). The infection of TRV- TCONS_00008447 into flowers aroused an emission increase of terpenoid 7,8-dihydro-β-ionone by 3.9 folds compared to TRV control flowers (**Figure 9D**). The qRT-PCR result revealed that the expression of TCONS_00008447 was decreased by 43% compared to the control flowers (**Figure 9C**). The result suggested that the TCONS_00008447 was involved in the regulation of floral scent production in the rose.

## Discussion

### Biosynthesis of floral scent was controlled developmentally in *R. hybrida*

Floral scent production markedly changes during flower development, corresponding with its role in attracting pollinators to plants (Shalit et al., 2003; Boatright et al., 2004; Colquhoun et al., 2010; Rodriguez-Saona et al., 2011). In common, flowers did not emit fragrance until they arrived at a state similar to EF stage of rose in this paper, and emissions of most of their fragrance compounds peaked at a state that the petals were opened and stamens were exposed thoroughly, which could be deemed as full-flowering (FF) stage. However, some fatty acid derivatives decreased from the EF stage to the end of the flower development (Shi et al., 2018; Yang et al., 2021).

*R. hybrida* was the hybrid progeny of Chinese and European roses, and inherited the complicated floral-scent profiles of the parents. During the flowering process of the rose, hundreds of volatile molecules could be obtained (Joichi et al., 2005; Bendahmane et al., 2013). Unlike other species, emissions of the most floral-scent compounds in the rose peaked at the SF but not FF stage (Guterman et al., 2002; Shalit et al., 2004; Feng et al., 2014; Yeon and Kim, 2020), indicating that the rose produced most of its scent compounds during the petal opening process. It was consistent with the second phase of petal development in the rose that petals grow rapidly resulting only from cell expansion, which was accompanied with the most production of floral scent (Guterman, 2002). In the research, emissions of the fatty acid derivatives peaked in SF stage as well and retained a higher level until the LF stage in ‘Tianmidemeng’, inconsistent with results in other species but consistent with former reports in roses (Shalit et al., 2004). Therefore, some different regulatory mechanisms may be involved in fatty acid derivatives production in rose. Whatever, the results also supported another finding that there was no direct relationship between fragrance synthesis and senescence of rose flowers (Borda et al., 2011).

### A reliable list of lncRNAs from rose flowers

Although many lncRNAs have been identified from numerous model plants, such as *Arabidopsis*, there are limited studies on lncRNAs in rose and further research is required in this area (Liu et al., 2022). In the present study, with the strand-specific RNA-Seq and a strict criteria pipeline widely used in previous studies in plants (Zhu et al., 2015; Zhang et al., 2016; Wang et al., 2017), a total of 13,957 lncRNAs were identified and classified in rose, a model plant for the study of floral scent (**Figure 3**). Common transcriptome library construction and sequencing cannot separate the sense and antisense strands, which resulted in quite a missing of lncRNAs. With application of the strand-specific RNA-Seq, the strand orientation information of the lncRNAs were conserved, thus facilitating their identification and functionally analysis (Di et al., 2014; Shuai et al., 2014). The development of third-generation sequencing technology further promoted the study for lncRNAs, for it could catch the strand orientation information without strand-specific library and obtain longer lncRNAs (Cui et al., 2019; Teng et al., 2019). Therefore, the limitations of our rose lncRNA list still remained, including that the pair-end sequencing could not obtain complete sequences for all lncRNAs thorouly and the RNA-seq was not deep enough to explore rose lncRNAs fully. In summary, the specific sequencing and strict bioinformatics criteria of the current study generated a relatively reliable list of rose lncRNAs, which will potentially benefit to other researchers.

### LncRNAs played a role in regulating floral scent synthesis in roses

Remarkable progress has been made in elucidating important roles of lncRNAs in multiple of physiological processes in plants, including phosphate homeostasis, vernalization response, immune response, root development, seedling photomorphogenesis, gametophyte development, stress response, nitrate response, rice yield, leaf morphological development, disease resistance, pathogen infection, tomato ripening process, formation of root nodules, pollen development, male fertility, and so on (Wu et al., 2020).

LncRNAs might be a general component of plant immune responses, for numerous differentially expressed lncRNAs were identified from the pathogen-infected plants including tomato, cotton, arabidopsis, rice, and mutants of some of them were shown to alter plant resistance to pathogens (Liu et al., 2022). For example, accumulation of a pathogen-responsive lncRNA ALEX1 could activate the (jasmonic acid) JA pathway in rice and enhance its resistance to bacterial blight (Yu et al., 2020), LincRNA CRIR1 regulated cold stress response of the cassava by modulating the expression of stress-responsive genes and increasing the translational yield (Li et al., 2022). It was found that lncRNAs could be transferred between *Cuscuta Parasites* and its host soybean plants, indicating their critical role as regulators to coordinate the host–dodder interaction (Wu et al., 2022). Moreover, lncRNAs could act as a switch in balancing plant defense and growth. In *Arabidopsis thaliana*, lcRNA SABC1 could repress plant immunity *via* decreasing transcription factor gene *NAC3* and *isochorismate synthase 1* (*ICS1*) transcriptions. However, upon pathogen infection, SABC1 was downregulated to depress plant resistance to bacteria and viruses (Liu et al., 2022). Due to the dual role in plant pollination and defense of floral scent, whether lncRNAs involved in its production functioned similarly was worth anticipating.

In spite of various roles of lncRNAs in plant physiological processes, their functions in floral scent synthesis were absent in current researches. By a comprehensive approach combining methods of differential-expression analyses, co-location and co-expression prediction and WGCNA analysis, we predicted candidate lncRNAs for floral production in the rose. The results of the consequent VIGS experiment initially confirmed their regulator role in rose floral scent synthesis. However, function mechanisms of them would be further investigated.

How lncRNAs regulate diverse biological processes is far from clear. It was found that lncRNAs with low expression tended to amplify their action by targeting transcription factors, while the cis-acting lncRNAs usually regulated the expression of their neighboring genes in the nucleus *via* epigenetic modifications (Gil and Ulitsky, 2020; Rinn and Chang, 2020). The trans-acting lncRNAs were usually indentified by a co-expressional grithm. In rice plants infected by rice black-streaked dwarf virus (RBSDV), a co-expression network of 56 differentially-expressed mRNAs and 20 differentially-expressed lncRNAs was construced, in which five mRNAs were verified to be regulated by three lncRNAs by the experiment conducted in rice calli (Zhang et al., 2020). Cis-acting LncRNAs functioned by recruiting DNA methyltransferases or demethylases to regulate the target gene transcription. In *Arabidopsi*, the lncRNA COLDAIR was generated from the intron of *FLC* and repress its expression by recruiting PRC2 *via* H3K27me3 (Heo and Sung, 2011).

### LncRNAs might regulate floral scent production *via* transcription factors

In the past decade, several TFs were found to play important role in floral scent synthesis. Several TFs regulate gene expression of phenylpropanoid/benzenoid production in flowers, including four R2R3-type MYB TFs in petunia—*ODO1* (Verdonk et al., 2005), *EOBI* (Van Moerkercke et al., 2011; Spitzer-Rimon et al., 2012), *EOBII* (Spitzer-Rimon et al., 2010; Colquhoun et al., 2011; Van Moerkercke et al., 2011), and *PH4* (Cna'ani et al., 2015)—and two repressor TFs—*PhMYB4* (Colquhoun et al., 2010) in petunia and *MYB3* in *Arabidopsis* (Zhou et al., 2017). For terpene biosynthesis, two cases of TFs have been reported in floral organs. Firstly, in *A. inflorescence*, bHLH-like transcription factor AtMYC2 promoted the synthesis of sesquiterpene (E)-β-caryophyllene by binding to *AtTPS11* and *AtTPS21* promoters of the terpene synthetase gene (Hong et al., 2012). Secondly, in petals of *Phalaenopsis bellina*, five TFs—PbbHLH4, PbbHLH6, PbbZIP4, PbERF1, and PbNAC1—promoted the synthesis of floral terpene components, with PbbHLH 4 improving the expression of geranyl diphosphate synthase gene *GDPS* by combining with its promoter and enhancing the synthesis of monoterpenes in floral scent (Chuang et al., 2018).

LncRNAs were supposed to target transcription factor genes to amplify their actions. A heat−inducible antisense lncRNA was involved in gametophyte development of *A. thaliana* by controlling the heat shock factor HSFB2a (Wunderlich et al., 2014). A novel ribonucleoprotein complex with lncRNA APOLO and the transcription factor WRKY42 forms a regulatory hub to trigger root hair cell expansion in response to cold by activating the master regulator RHD6 in *Arabidopsis* (Moison et al., 2021). In *P. tomentosa*, lncRNA PMAT interacted epistatically with *PtoMYB46* promoted Pb^2+^ tolerance, uptake and plant growth of poplar by repressing *PtoMATE* and *PtoARF2* (Chen et al., 2022). Interestingly, lncRNAs tended to target transcription factor genes nearby them, such as TWISTED LEAF, circular RNA (circRNA) SEP3, and SUF (Conn et al., 2017; Liu et al., 2018; Hisanaga et al., 2019). In the present study, homolog transcripts of 169 TFs reported to be involved in the production of floral volatile compounds in plants were obtained from rose RNA-seq data and were predicted to be target genes of 208 lncRNAs. Whether and how the lncRNAs regulate their expression would be further verified by biological experiments in the rose.

LncRNAs could bind miRNAs as eTMs to regulate the expressions of target mRNAs. The LnRNA TCONS_00021861 could regulate YUCCA7 by sponging miR528-3p, to activate IAA biosynthetic pathway and confer resistance to drought stress in rice (Chen et al., 2021). Overexpression of lncRNA23468 in tomato significantly decreased expression of miR482b, and then increased the expression of its target genes *NBS-LRRs*, resulting in enhanced resistance to *Phytophthora Infestans* (Jiang et al., 2019). LncRNA regulated the expression of *CSD1* by indirectly through competitively binding miR398 to improve cold resistance of winter wheat (Lu et al., 2020). In the rose, transcriptomic sequencing revealed the presence of a large number of ncRNAs, and the miR156 was proposed to be involved in synthesis of some terpenes in petals (Raymond et al., 2018). Although the prediction of target miRNAs for lncRNAs were lacked in this paper, it was essential to supplement for it in the future. Some protocols have been developed for miRNA–lncRNA interaction prediction in plants, such as an ensemble deep learning model based on multi-level information enhancement and greedy fuzzy decision (PmliPEMG), which could be applied to the cross-species prediction (Kang et al., 2021), an ensemble pruning protocol that for minining plant eTMs by predicting miRNA-lncRNA interactions based on dual-path parallel ensemble pruning method (Kang et al., 2022). By constructing the lncRNA-miRNA-mRNA regulatory network through biological experiment, the functions of potential eTMs could be further inferred through enrichment analysis.

## Conclusions

An overview of the transcriptional regulation of floral scent production by lncRNAs in rose flowers was generated using a variety of techniques and analyses including genome-wide identification, characterization, differential expression, and co-expression network analysis of intergenic/intronic lncRNAs. As the first lncRNA research in rose, 13,957 lncRNAs were identified, including 10,887 annotated lncRNAs and 3070 novel lncRNAs, while 19 core lncRNAs were predicted to be candidates participating in floral scent synthesis. WGCNA suggested that expression of the 11 lncRNAs is highly enriched in co-expressed modules that are related to floral scent synthesis pathways, and function of one of them were confirmed by the VIGS experiment. Future research efforts will aim to elucidate the mechanism by which these lncRNAs regulate floral scent production. Overexpression, RNA interference, and promoter analysis are useful experimental approaches for characterizing lncRNA functions, which might provide valuable information for improving floral scent in rose.

## Data availability statement

The original contributions presented in the study are publicly available. This data can be found here: NCBI, PRJNA667625.

## Author contributions

ZZ: conceived and designed the experiments. ZZ, SS, JW and XL: methodology. SS and SZ: experiments. SS: analysis of data. SS and SZ: writing original draft preparation. ZZ and SS: writing—review and editing. ZZ: supervision. All authors contributed to the article and approved the submitted version.

## Funding

This work was funded by Shandong Provincial Natural Science Foundation (Grant number ZR2020QC159), Natural Science Foundation of Beijing Municipality (Grant number 6222030), National Natural Science Foundation of China (Grants numbers 32102430, 31501791, 31772344 and 31972444) and Innovation Project of Shandong Academy of Agricultural Sciences (Grants numbers CXGC2021B17, CXGC2022A06).

## Acknowledgments

The authors acknowledge Pro. Mignfang Yi for her scientific suggestions, acknowledge Novogene Co., Ltd. for their technical support, and acknowledge Researcher Yumeng Huo for providing server for our bioinformatic analysis.

## Conflict of interest

The authors declare that the research was conducted in the absence of any commercial or financial relationships that could be construed as a potential conflict of interest.

## Publisher’s note

All claims expressed in this article are solely those of the authors and do not necessarily represent those of their affiliated organizations, or those of the publisher, the editors and the reviewers. Any product that may be evaluated in this article, or claim that may be made by its manufacturer, is not guaranteed or endorsed by the publisher.

## Supplementary material

Supplementary Figure 1 qRT-PCR validation of selected lncRNAs expression across flowering stages in rose ‘Tianmidemeng’. Expression levels of selected transcripts measured by qRT-PCR and RNA-Seq are showed in the same histograms. Black columns indicate relative gene expression levels detected by qRT-PCR (left y-axis; normalized units). Grey columns represent expression determined by RNA-Seq in RPKM units (right y-axis).

Supplementary Figure 2 Associations between module and floral volatiles by WGCNA analysis. Each row corresponds to a module eigengene, column to a trait. Each cell contains the corresponding correlation and p-value. The table is color-coded by correlation according to the color legend.
