# Supplementary material for: Identification of long non-coding RNAs involved in floral scent of Rosa hybrida
Source: Front Plant Sci. 2022 Oct 4;13:996474. doi: 10.3389/fpls.2022.996474 (PMC9577252; doi:10.3389/fpls.2022.996474)
Supplement: Supplementary Figure 1 — qRT-PCR validation of selected lncRNAs expression across flowering stages in rose ‘Tianmidemeng’. Expression levels of selected transcripts measured by qRT-PCR and RNA-Seq are showed in the same histograms. Black columns indicate relative gene expression levels detected by qRT-PCR (left y-axis; normalized units). Grey columns represent expression determined by RNA-Seq in RPKM units (right y-axis). [file DataSheet_1.zip › Supplementary files/Supplementary Table 1. Primers used in qRT-PCR detection and vector construction..docx]

| **Genes** | **Primer name** | **Sequence （5’-3’）** |
| --- | --- | --- |
| TCONS_00008447 | q8447-F | ACATCGAGACTCCCTGTTCC |
|  | q8447-R | AAACCTTCAGCCATGAATTCGG |
| TCONS_00034955 | q4955-F | GAAGAATAAGCCATGAAGCCTCC |
|  | q4955-R | TAGAATTACACCATGAAGGGAGTG |
| XR_002924185.1 | q4185-F | TCGTATTTGGTGCGGTGTCT |
|  | q4185-R | CGTTGCCTCCGTCAAATGAG |
| TCONS_00111355 | q1355-F | GGCCTAAACCCCTTACTGGT |
|  | q1355-R | ATGAACGTTGTCCCCTTCCT |
| XR_002931444.1 | q1444-F | CGAAAACCCGTTTCCCCATT |
|  | q1444-R | GCTACCCCATCACTCGTGG |
| TCONS_00081546 | q1546-F | TCTCAGTTTCAGTGGCCCTT |
|  | q1546-R | AGCAGAATCCCCACTATGGC |
| TCONS_00127824 | q7824-F | CCCAATTCTCAAGTACATCAAG |
|  | q7824-R | TTGCAATGGATGGTAACACT |
| XR_002925539.1 | q5539-F | ACTACAGAATCAAGCAAGGTCTCT |
|  | q5539-R | TGAATTGACTCTATCCAAGACCA |
| XR_002929995.1 | q9995-F | AGACGTACTTGGTCTTGCTG |
|  | q9995-R | CACCAAAGTGGTTGGCTTCA |
| XR_002930104.1 | q0104-F | ACCCACGAAATAGGCTTGGTT |
|  | q0104-R | ATCGTTTCCTCCCCAAACCC |
| *GAPDH* | GAPDH-F | ATCCATTCATCACCACCGACTACA |
|  | GAPDH-R | GCATCCTTACTTGGGGCAGAGA |
| TCONS_00008447 | V8447-F | GGGGACAAGTTTGTACAAAAAAGCAGGCTAGGGTA  GCTGTTTCAGTCCC |
|  | V8447-R | GGGGACCACTTTGTACAAGAAAGCTGGGTTTCAGC  CATGAATTCGGAGACA |
