# Supplementary figures and images for: Identification of long non-coding RNAs involved in floral scent of Rosa hybrida
Source: Front Plant Sci. 2022 Oct 4;13:996474. doi: 10.3389/fpls.2022.996474 (PMC9577252; doi:10.3389/fpls.2022.996474)

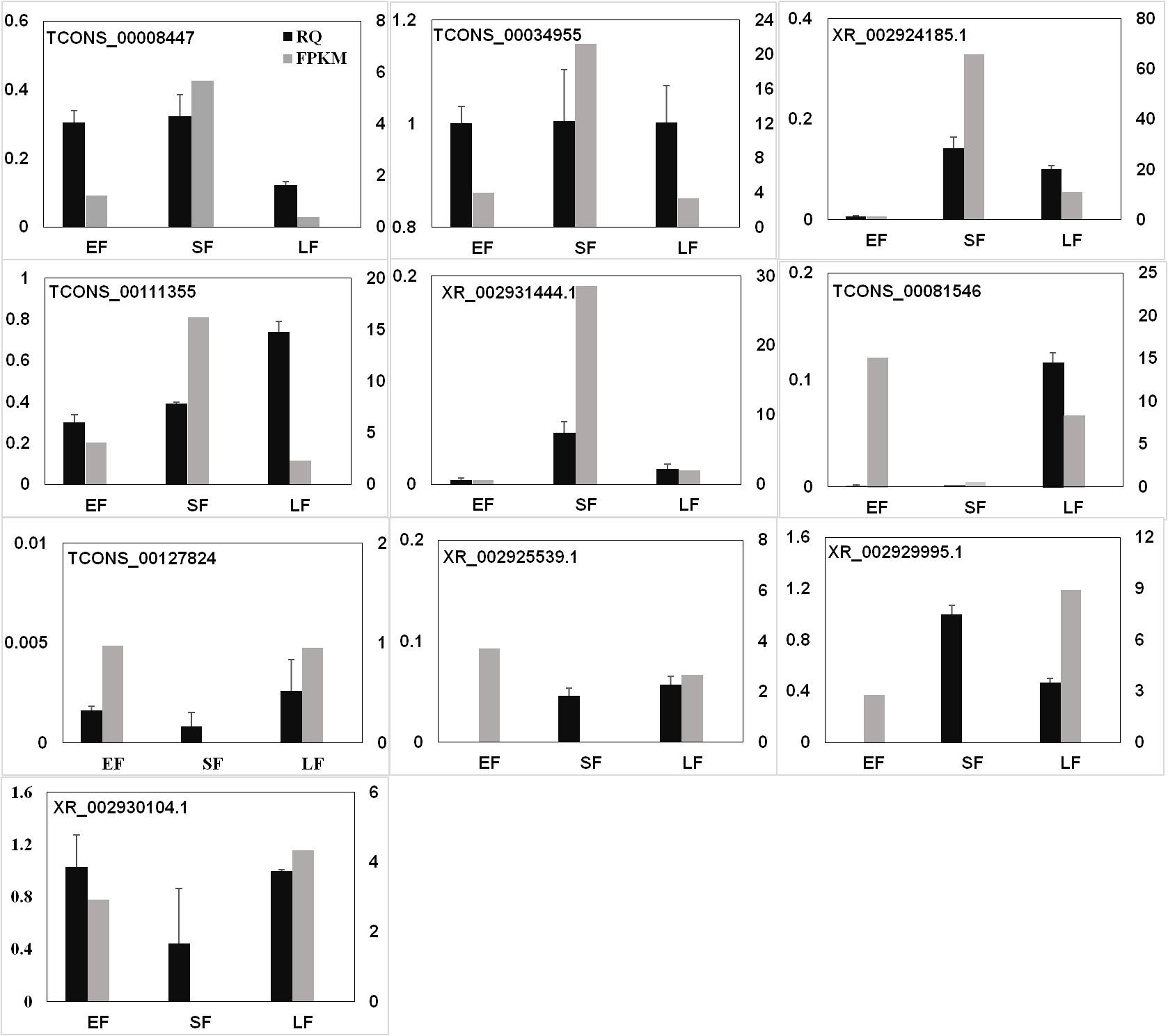

Supplement: Supplementary Figure 1 — qRT-PCR validation of selected lncRNAs expression across flowering stages in rose ‘Tianmidemeng’. Expression levels of selected transcripts measured by qRT-PCR and RNA-Seq are showed in the same histograms. Black columns indicate relative gene expression levels detected by qRT-PCR (left y-axis; normalized units). Grey columns represent expression determined by RNA-Seq in RPKM units (right y-axis). [file DataSheet_1.zip › Supplementary files/Supplementary Figure 1. qRT-PCR validation of selected lncRNAs expression across flowering stages in rose í«Tianmidemengí»..jpg]
